# Learning curve analysis across three surgical eras in Ivor Lewis esophagectomy

**DOI:** 10.1007/s11701-026-03327-3

**Published:** 2026-03-30

**Authors:** Mazen A. Juratli, Franziska Viola Damhorst, Ann-Kathrin Eichelmann, Jennifer Merten, Nader El-Sourani, Mazen Aldarwish, Andreas Pascher, Jens Peter Hoelzen

**Affiliations:** Department of General, Visceral and Transplant Surgery, University Hospital Muenster, University of Muenster, Albert-Schweitzer-Campus 1, 48149 Muenster, Germany

**Keywords:** Robot-assisted minimally invasive esophagectomy, Learning curve, Surgical evolution, CUSUM analysis, Esophageal cancer surgery

## Abstract

Comprehensive learning curve analyses across the evolution from open to robot-assisted esophagectomy remain limited. This retrospective single-center study analyzed 376 consecutive Ivor Lewis esophagectomies (2012–2023) across three eras: open (*n* = 127), hybrid-open (*n* = 52), and robot-assisted minimally invasive esophagectomy (RAMIE, *n* = 197). Learning curves were assessed using CUSUM analysis, and patient-specific factors were evaluated to guide case selection. Open esophagectomy reached plateau at case 65 with subsequent outcome deterioration. Hybrid-open achieved early plateau (case 15) but showed fluctuating metrics. RAMIE demonstrated steady improvement with CUSUM-derived stabilization achieved at cases 45–50 (hybrid-RAMIE) and 70 (total RAMIE), showing significant reductions in operative times, hospital stay, and ICU stay (*p* < 0.001). Higher BMI, advanced nodal disease, and comorbidity burden negatively impacted outcomes. In our center, CUSUM-derived stabilization was observed after approximately 45–50 cases for hybrid-RAMIE and about 70 cases for total RAMIE. Strategic case selection—prioritizing lower BMI, limited nodal burden, and minimal comorbidities may accelerate early stabilization of performance. High-volume centers may benefit from total-robotic approaches, while lower-volume programs may favor hybrid pathways.

**Supplementary Information:**

The online version contains supplementary material available at 10.1007/s11701-026-03327-3.

## Introduction

Esophageal carcinoma ranks among the most aggressive malignancies worldwide, with surgical esophagectomy remaining the cornerstone of curative treatment despite significant perioperative morbidity [1–4]. The evolution of surgical techniques has fundamentally transformed the operative landscape, progressing from open procedures through hybrid approaches to contemporary robot-assisted minimally invasive esophagectomy (RAMIE) [5, 6].

This study documents the institutional evolution of Ivor Lewis esophagectomy across three epochs: open (2012–2017), hybrid-open (2014–2018), and robot-assisted minimally invasive esophagectomy (RAMIE, 2018–2023), mirroring global transitions demonstrating progressive outcome improvements [7–13].

Focusing exclusively on the Ivor Lewis technique within a consistent institutional framework provides controlled assessment of technological evolution [14–17] across surgical epochs.

Learning curves were assessed using cumulative sum (CUSUM) analysis for continuous parameters and cumulative frequency analysis for binary outcomes.

The study addresses three objectives: comparing learning curves across surgical epochs, evaluating hybrid-robotic versus total robotic RAMIE approaches, and identifying patient-specific factors influencing learning parameters. While multicenter studies demonstrate superior outcomes with total robotic esophagectomy [9, 18, 19], comparative learning curve analyses examining the transition between these robotic approaches remain limited. This learning curve comparison addresses the practical question: Does the transition from hybrid-robotic to total robotic technique justify the additional complexity and resource investment?

This analysis provides practical guidance for centers implementing robotic surgery while addressing whether technological advancement consistently improves learning trajectories. While robotic surgery has been associated with favorable perioperative outcomes [8, 20], extended learning trajectories raise implementation considerations for centers with varying case volumes [14, 21].

## Materials and methods

### Study design

This retrospective single-center study compared learning curves across open, hybrid-open, and RAMIE approaches, evaluated hybrid-robotic versus total robotic RAMIE, and identified patient-specific factors influencing learning parameters.

### Setting and surgical experience

The study was conducted at University Hospital Münster. A total of 376 esophageal resections (January 2012–May 2023) were analyzed.

All key operative steps were performed by attending surgeons; critical steps were not delegated to trainees. Open and hybrid-open esophagectomies were performed by a single experienced general surgeon with extensive prior experience in upper gastrointestinal surgery. Robot-assisted minimally invasive esophagectomies (RAMIE) were performed by a single general surgeon who had accumulated extensive experience in upper gastrointestinal surgery since 2008. Prior to initiating RAMIE procedures, this surgeon had completed approximately 100 robotic fundoplications over 16 months. To support the implementation phase, experienced proctors were present during the first and sixth RAMIE procedures.

### Learning curve definition and parameters

We defined the learning curve as the number of operations required for a surgeon to achieve stable performance levels. Our learning curve assessment analyzed the following parameters: operative duration, postoperative hospital length of stay, postoperative intensive care unit stay, occurrence of severe postoperative complications (Clavien-Dindo ≥3b) [22, 23], anastomotic leakage, and postoperative pneumonia.

### Patient population

This retrospective study included all adult patients (≥ 18 years) with histologically confirmed, resectable intrathoracic or abdominal esophageal carcinoma at diagnosis. All cases employed the Ivor Lewis procedure. Exclusion criteria included patients with cervical esophageal carcinoma or gastroesophageal junction carcinomas with infiltration of the gastric cardia (Siewert Type III). Patients whose operations were terminated due to intraoperatively determined inoperability were also excluded (*n* = 3). The final analysis encompassed 376 esophageal resections performed between January 2012 and May 2023. Open esophagectomies (*n* = 127) were performed between January 2012 and November 2017. Hybrid-open esophagectomies (*n* = 52) were conducted between June 2014 and April 2018. Robot-assisted minimally invasive esophagectomies (RAMIE, *n* = 197) were performed from December 2018 to May 2023. Of these 197 RAMIE procedures, 76 were performed as hybrid-robotic procedures using the da Vinci Si Robot System between December 2018 and December 2020. From January 2021 to May 2023, 121 total robotic procedures were performed using the da Vinci Xi Robot System. A total of 325 patients received neoadjuvant therapy using either CROSS (ChemoRadiotherapy for Oesophageal cancer followed by Surgery Study) protocol or FLOT (Fluorouracil, Leucovorin, Oxaliplatin, and Docetaxel) protocol chemotherapy, or combined radiochemotherapy.

### Surgical technique

All procedures employed the standardized Ivor Lewis esophagectomy technique consisting of sequential abdominal and thoracic phases with gastric conduit creation, regional lymphadenectomy, and intrathoracic end-to-side esophagogastrostomy [24]. The open procedure was performed via upper midline laparotomy and right posterolateral thoracotomy. Hybrid-open technique combined laparoscopic abdominal mobilization with open thoracotomy. Hybrid-RAMIE employed laparoscopic abdominal phase followed by robotic thoracic resection using the da Vinci Si system. Total robot-assisted minimally invasive esophagectomy (RAMIE) was performed using the da Vinci Xi system following standardized protocols, with both abdominal and thoracic phases completed robotically. Detailed technical aspects have been published previously [20]. Briefly, the intrathoracic esophagogastric anastomosis was performed using a circular stapler (EEA 25–29 mm) with partial hand-sewn reinforcement and fat tissue wrapping. A Kocher maneuver and pyloroplasty procedures were not routinely performed. Feeding jejunostomy was placed only in selected patients. Preemptive endoluminal vacuum therapy (EndoVAC) was used routinely as part of our institutional leak-prevention strategy across all surgical epochs.

### Postoperative care

Postoperative management followed standardized institutional protocols with initial intensive care unit (ICU) admission, early mobilization, and gradual oral intake progression. Through mid-2018, patients were managed on a surgery-dedicated ICU with extended monitoring. From mid-2018, institutional reorganization under new clinical leadership transitioned care to centralized interdisciplinary ICU structures with earlier step-down protocols. Esophagogastroduodenoscopy was performed on postoperative day 5 to assess anastomotic integrity, with EndoVAC removal if no abnormalities were detected.

### Endpoints

Perioperative and short-term postoperative parameters were retrospectively assessed from patient records. Key parameters included total operative duration (skin-to-skin time in minutes), postoperative hospital and intensive care unit length of stay (days), occurrence of severe postoperative complications (Clavien-Dindo ≥3b), anastomotic leakage, and postoperative pneumonia. Anastomotic leakage was recorded using a surveillance-based, sensitive definition. In addition to clinically or radiologically evident leaks (e.g. contrast extravasation), routine endoscopy on postoperative day 5 (performed in the context of EndoVAC assessment) identified anastomotic abnormalities suggestive of impaired healing (e.g. widening, exposed staples, friable tissue, fibrin deposition), which were also documented as leaks even in the absence of clinical symptoms or radiologic confirmation. Accordingly, our definition extends beyond the Esophagectomy Complications Consensus Group (ECCG) criteria and may therefore result in higher reported leak rates than studies restricted to ECCG-defined full-thickness defects or contrast extravasation.

For precise comparison between hybrid-RAMIE and total RAMIE techniques, separate analysis of abdominal and thoracic operative durations was performed.

To identify optimal “training cases” for inexperienced RAMIE surgeons, the following patient characteristics were analyzed for their influence on key parameters: age, gender, body mass index (BMI), American Society of Anesthesiologists (ASA) Score, World Health Organization (WHO) performance status, Charlson comorbidity index, antihypertensive medication, steroid or immunosuppressive therapy, anticoagulation, tumor location and histology, preoperative tumor and lymph node status, presence of metastasis (preoperative M-status) and neoadjuvant therapy use. The Results and Discussion for this exploratory “training cases” analysis are reported in the Supplementary Material.

### Statistical analysis

#### Learning curve analysis

Two validated methodologies were used to assess learning curves for continuous and binary parameters.

CUSUM Analysis was applied for continuous outcomes (operative duration, hospital length of stay, ICU stay). CUSUM analysis has been extensively validated for learning curve assessment in surgery, providing objective, case-by-case evidence of performance changes [25–28]. The CUSUM value for case *n* was calculated as the cumulative sum of the deviations of each individual case value (*xi*) from the overall mean (*µ*) of the respective group (CUSUMn = Σ(xi - µ)) [14, 29]. A plateau in the curve followed by a descent indicates stabilization relative to the cohort mean.

Cumulative Frequency Analysis was used for binary parameters (severe complications, anastomotic leakage, postoperative pneumonia). This method visualizes the cumulative count of observed events over the chronological case series compared to the statistically expected number of events, which is based on the group’s overall mean event rate.

#### Phase-based analysis and predictive factors

For a detailed comparison of the robotic techniques, the learning curves for hybrid-RAMIE and total RAMIE were divided into three sequential, equal-sized phases: an “Initial Phase,” a “Consolidation Phase,” and an “Expert Phase”. Here, ‘expert phase’ is used as an analytic label for the later phase and does not imply surgical mastery. Phase boundaries were predefined as equal case thirds for comparability. The average complication rate for each phase was calculated and displayed as bar charts. To identify significant improvements, performance in the consolidation phase was compared to the expert phase. Differences were tested using t-tests for continuous variables and pairwise chi-square tests for binary variables.

To identify patient-specific factors influencing key outcomes, linear regression analysis was performed for continuous variables and multivariate logistic regression for binary endpoints. To maintain practical clinical applicability for risk stratification, only linear main effects were considered without modeling interaction terms.

Statistical analysis was performed using SPSS (Version 30.0.0; IBM Corp., Armonk, NY) and Python (Version 3.12). In Python, data management was handled with the Pandas library, statistical tests were conducted with SciPy, and all visualizations (including CUSUM and cumulative frequency analyses) were generated using Matplotlib. A p-value < 0.05 was considered statistically significant.

Due to the retrospective, non-randomized comparison of three surgical eras, selection bias, confounding, and an era effect are possible. We included all consecutive eligible patients using predefined exclusion criteria. From December 2018 onward, RAMIE was the default approach for all eligible Ivor Lewis cases at our center. Outcomes and covariates were obtained from routine clinical documentation using standardized definitions where available.

All variables used in the analyses were complete, with no missing values.

### Ethical considerations

Ethical approval was obtained from the Ethics Committee of the Medical Association of Westphalia-Lippe and the University of Münster (reference: 2022-123-f-S). The study was conducted at a certified acute care level 1 academic hospital. Race and ethnicity distributions were not collected as this information was not structurally recorded as part of routine patient data.

## Results

A total of 376 consecutive patients underwent Ivor Lewis esophagectomy during the study period. Patient demographics and baseline characteristics are summarized in Table 1. Across the three surgical approaches, most baseline features were broadly comparable. However, patients in the RAMIE cohort presented with slightly more favorable preoperative status—reflected by a better WHO performance distribution, a difference in comorbidity burden and a higher proportion of N0 disease—than those in the open and hybrid-open groups. These imbalances may suggest non-random case selection and introduce potential confounding; we therefore performed multivariable risk-adjusted analyses for the RAMIE vs. Open comparison (see ‘Risk-Adjusted Outcomes’ and Supplementary Table S1).

**Table 1:** Baseline Characteristics (*n* = 376)

	Open (*n* = 127) n (%)	Hybrid-Open (*n* = 52) n (%)	RAMIE (*n* = 197) n (%)	*p* value
*Age, years*				0.145
< 50	15 (11.8)	6 (11.5)	7 (3.6)	
50–59	30 (23.6)	14 (26.9)	46 (23.4)	
60–69	53 (41.7)	19 (36.5)	83 (42.1)	
70–79	26 (20.5)	11 (21.2)	49 (24.9)	
≥ 80	3 (2.4)	2 (3.8)	12 (6.1)	
*Gender*				0.407
Female	28 (22.0)	8 (15.4)	33 (16.8)	
Male	99 (78.0)	44 (84.6)	164 (83.2)	
*Ethnicity*				
White	127 (100)	52 (100)	197 (100)	
*BMI*,* kg/m*^*2*^				0.720
<20	16 (12.6)	5 (9.6)	16 (8.1)	
20–30	88 (69.3)	38 (73.1)	140 (71.1)	
>30	23 (18.1)	9 (17.3)	41 (20.8)	
*ASA score*				0.191
1	6 (4.7)	2 (3.8)	6 (3.0)	
2	76 (59.8)	34 (65.4)	99 (50.3)	
3	43 (33.9)	16 (30.8)	91 (46.2)	
4	2 (1.6)	0 (0.0)	1 (0.5)	
*WHO performance status*				< 0.001
1	9 (7.1)	6 (11.5)	71 (36.0)	
2	57 (44.9)	23 (44.2)	96 (48.7)	
3	58 (45.7)	22 (42.3)	27 (13.7)	
4	3 (2.4)	1 (1.9)	3 (1.5)	
*Type of carcinoma*				0.353
Adenocarcinoma	99 (78.0)	42 (80.8)	166 (84.3)	
Squamous cell carcinoma	28 (22.0)	10 (19.2)	31 (15.7)	
*Locations of tumor*				0.689
Upper third	3 (2.4)	1 (1.9)	1 (0.5)	
Middle third	11 (8.7)	4 (7.7)	16 (8.1)	
Lower third & Gastroesophageal junction	113 (89)	47 (90.4)	180 (91.4)	
*Neoadjuvant therapy*				0.216
Chemotherapy	30 (23.6)	13 (25.0)	63 (32.0)	
Chemoradiotherapy	75 (59.1)	34 (65.4)	105 (53.3)	
Radiotherapy	0 (0.0)	0 (0.0)	5 (2.5)	
None	22 (17.3)	5 (9,6)	24 (12.2)	
*Charlson Comorbidity Index*				0.013
2	14 (11.0)	4 (7.7)	4 (2.0)	
3	21 (16.5)	12 (23.1)	31 (15.7)	
4	42 (33.1)	16 (30.8)	49 (24.9)	
5	32 (25.2)	10 (19.2)	47 (23.9)	
6	11 (8.7)	6 (11.5)	30 (15.2)	
7	6 (4.7)	3 (5.8)	16 (8.1)	
8	0 (0.0)	1 (1.9)	10 (5.1)	
9	1 (0.8)	0 (0.0)	2 (1.0)	
10	0 (0.0)	0 (0.0)	3 (1.5)	
11	0 (0.0)	0 (0.0)	5 (2.5)	
*Pretherapeutic T-status*				0.079
T1	10 (7.9)	5 (9.6)	24 (12.2)	
T2	37 (29.1)	19 (36.5)	36 (18.3)	
T3	79 (62.2)	28 (53.8)	133 (67.5)	
T4	1 (0.8)	0 (0.0)	4 (2.0)	
*Pretherapeutic N-status*				< 0.001
N0	20 (15.7)	11 (21.2)	92 (46.7)	
N+	107 (84.3)	41 (78.8)	105 (53.3)	
*Pretherapeutic M-status*				0.388
M0	125 (98.4)	51 (98.1)	189 (95.9)	
M+	2 (1.6)	1 (1.9)	8 (4.1)	
*Antihypertensive therapy*	48 (37.8)	17 (32.7)	120 (60.9)	< 0.001
*Cortisone therapy*	0 (0.0)	0 (0.0)	5 (2.5)	0.100
*Immunosuppressive therapy*	0 (0.0)	0 (0.0)	2 (1.0)	0.401
*Anticoagulation*	11 (8.7)	2 (3.8)	60 (30.5)	< 0.001

*BMI* body mass index, *ASA* American Society of Anesthesiologists, *WHO* World Health Organization

As shown in Table 2, RAMIE exhibited a more favorable perioperative profile: significantly lower blood loss, shorter ICU and overall hospital stay, and lower rates of anastomotic leak and pneumonia, despite a longer operative time. The R0 resection rate was significantly higher with RAMIE, whereas 30-day mortality was low across all groups without a significant difference. Although the overall Clavien–Dindo distribution did not reach statistical significance (*p* = 0.079), there was a visible trend toward fewer higher-grade complications in the RAMIE cohort.

**Table 2:** Operative and postoperative outcomes (*n* = 376)

	Open (*n* = 127)	Hybrid-Open (*n* = 52)	RAMIE (*n* = 197)	*p* value
Operating time, median min (range)	300 (271–335)	321 (297–352)	424 (374–514)	< 0.001
*Postoperative hospital stay*,* median days (range)*	23 (16–45)	18 (15–34)	17 (13–25)	< 0.001
*ICU stay*,* median days (range)*	10 (7–20)	7 (5–17)	2 (1–4)	< 0.001
*Complications (Clavien-Dindo classification)*				0.079
CD 0	33 (26.0%)	21 (40.4%)	87 (44.2%)	
CD 1	10 (7.9%)	2 (3.8%)	18 (9.1%)	
CD 2	23 (18.1%)	10 (19.2%)	25 (12.7%)	
CD 3a	38 (29.9%)	12 (23.1%)	45 (22.8%)	
CD 3b	8 (6.3%)	3 (5.8%)	14 (7.1%)	
CD 4	5 (3.9%)	1 (1.9%)	4 (2.0%)	
CD 5	10 (7.9%)	3 (5.8%)	4 (2.0%)	
*Severe complications (CD* ≥ 3b)	23 (18.1%)	7 (13.5%)	22 (11.2%)	0.209
*Anastomotic leak*	45 (35.4%)	12 (23.1%)	33 (16.8%)	< 0.001
*Pneumonia*	37 (29.1%)	13 (25.0%)	29 (14.7%)	0.006
*Blood loss*,* median ml (range)*	500 (200–800)	75 (50–200)	50 (50–200)	< 0.001
*Lymph nodes harvested*	25 (17–49)	33 (26–37)	30 (25–37)	< 0.001
*Radicality* *of surgery*				0.002
R0	117 (92.1%)	43 (82.7%)	190 (96.4%)	
R1	10 (7.9%)	9 (17.3%)	7 (3.6%)	
*30-day-mortality*	1 (0.8%)	1 (1.9%)	0 (0.0%)	0.211

*ICU* Intensive Care Unit, *CD* Clavien-Dindo

### Risk-adjusted outcomes (RAMIE vs. Open)

To account for baseline imbalances, we performed multivariable regression analyses for the RAMIE vs. Open comparison, adjusting for age, BMI, WHO performance status, and Charlson Comorbidity Index. The hybrid-open cohort was not included in this adjusted comparative model because of its limited sample size, which could compromise model robustness, particularly for binary endpoints. After adjustment, RAMIE remained associated with shorter postoperative hospital stay (adjusted β = −16.7 days, *p* < 0.001) and ICU stay (adjusted β = −15.2 days, *p* < 0.001). The association with major complications (Clavien–Dindo ≥ 3b) was directionally favorable but did not reach statistical significance (adjusted OR = 0.51, *p* = 0.083). Detailed model results are provided in Supplementary Table S1.

### Evolution: learning curve evolution across surgical epochs

**Fig. 1:**
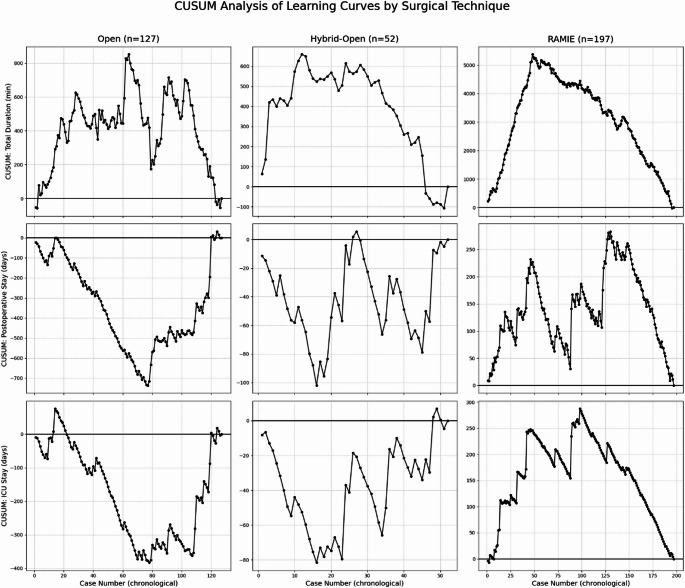
CUSUM learning curve profiles for total operative time, postoperative hospital stay, and ICU stay across chronological case order in the Open, Hybrid-Open, and RAMIE cohorts

**Fig. 2:**
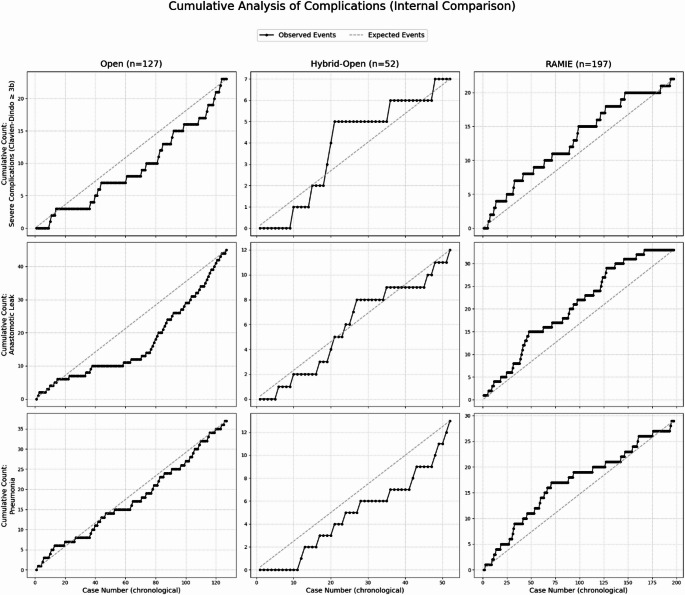
Cumulative Analysis of Severe Complications (Clavien–Dindo ≥3b), Anastomotic Leak, and Pneumonia across chronological case order in the Open, Hybrid-Open, and RAMIE cohorts

Figures 1 and 2 summarize the temporal performance across surgical approaches—Fig. 1 shows CUSUM learning-curve profiles for operative time, postoperative hospital stay, and ICU stay, while Fig. 2 presents cumulative complication plots (severe complications, anastomotic leak, pneumonia) against group-specific expected rates for the open, hybrid-open, and RAMIE cohorts.

#### Open esophagectomy

Operative duration plateaued at case 65 (Fig. 1). From case 70 onward, postoperative stay, ICU stay, and complications (Clavien-Dindo ≥3b, anastomotic leak, pneumonia) increased substantially.

#### Hybrid-open esophagectomy

Operative duration plateaued at case 15 (Fig. 1). Postoperative stay and ICU duration showed fluctuating patterns without clear trends. Complications decreased from case 20–25 but increased slightly after case 45.

#### Robot-assisted minimally invasive esophagectomy (RAMIE)

Operative duration plateaued at case 60 (Fig. 1). Postoperative stay and ICU duration improved after case 45, with temporary increases around case 76. Complications decreased after case 100, anastomotic leaks after case 45, and pneumonia after case 70.

### Robotics: learning curve comparison between hybrid-robotic and total robotic techniques

**Fig. 3:**
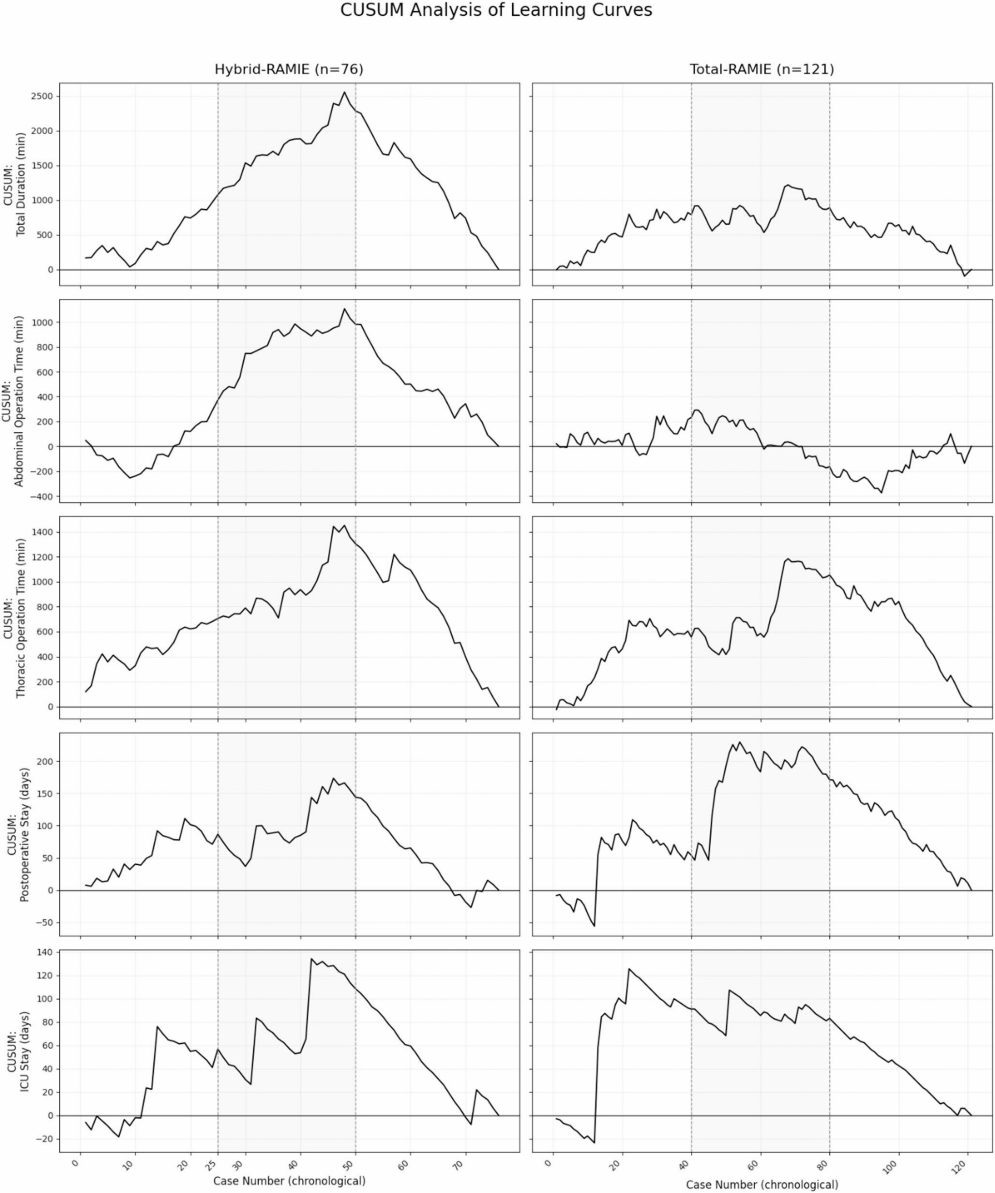
CUSUM learning-curve profiles for Hybrid-RAMIE and Total-RAMIE, showing total, abdominal, and thoracic operative time and postoperative hospital and ICU stay across chronological case order; vertical dashed lines demarcate initial, consolidation, and expert phases. *Hybrid-RAMIE*: CUSUM curves indicate a decline in total operative duration from ~case 47 onward; consolidation vs. expert phase showed a significant reduction (t = 4.1947, *p* < 0.001). Abdominal duration decreased from ~case 47 (t = 6.5091, *p* < 0.001), and thoracic duration from ~case 45 (t = 2.4047, *p* < 0.05). Hospital length of stay decreased from ~case 45 with a pronounced decline from ~case 50; consolidation vs. expert phase was significantly shorter (t = 4.4510, *p* < 0.001). ICU length of stay declined from ~case 45 and was significantly reduced in the expert phase (t = 3.5784, *p* < 0.001). *Total RAMIE*: Total operative duration declined from ~case 70; consolidation vs. expert phase showed a highly significant reduction (t = 10.0569, *p* < 0.001). Abdominal duration decreased from ~case 40 (most pronounced between cases 70–95) with a slight late increase after ~case 95; consolidation vs. expert phase remained significantly shorter (t = 5.8868, *p* < 0.001). Thoracic duration declined from ~case 70 (t = 5.0102, *p* < 0.001). Hospital length of stay decreased from ~case 70 (t = 6.9157, *p* < 0.001), and ICU length of stay from ~case 50 (t = 11.5463, *p* < 0.001)

**Fig. 4:**
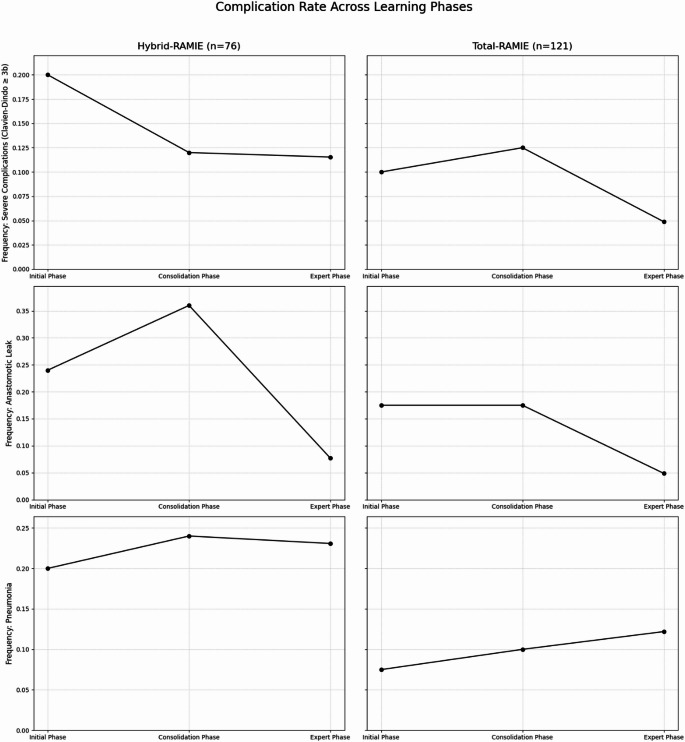
Complication rates across learning phases for Hybrid-RAMIE and Total-RAMIE, showing severe complications (Clavien–Dindo ≥3b), anastomotic leak, and pneumonia across the initial, consolidation, and expert phases. *Hybrid-RAMIE*: The rate of major complications (Clavien–Dindo ≥ 3b) decreased slightly from the initial to the consolidation phase and remained low in the expert phase; no difference was observed between consolidation and expert phases (X² = 0.0000, *p* = 1.0000). Anastomotic leakage increased in the consolidation phase compared with the initial phase, followed by a significant reduction in the expert phase (consolidation vs. expert: X² = 4.4798, *p* < 0.05). Postoperative pneumonia showed only minor fluctuations without a clear downward trend; consolidation vs. expert phases did not differ (X² = 0.0000, *p* = 1.0000). *Total RAMIE*: Major complications showed only minor fluctuations across phases, with a tendency toward lower rates in the expert phase; consolidation vs. expert phases did not differ significantly (X² = 0.7364, *p* = 0.3908). Anastomotic leakage remained nearly unchanged from the initial to the consolidation phase, with a visible decline in the expert phase that did not reach significance (consolidation vs. expert: X² = 2.2235, *p* = 0.1359). Postoperative pneumonia rates were largely stable across phases, with a slight increase from consolidation to expert phase; no significant difference was observed (X² = 0.0000, *p* = 1.0000)

Figures 3 and 4 show the learning curve assessment in detail: Fig. 3 shows CUSUM profiles for hybrid-RAMIE and total RAMIE with phase divisions (initial, consolidation, and expert phase) and Fig. 4 gives phase-specific rates of major complications (Clavien-Dindo ≥3b), anastomotic leak, and pneumonia.

Detailed phase-wise statistical comparisons (t/χ² and p values) are provided in the figure legends (Figs. 3 and 4).

#### Hybrid-RAMIE analysis

For the analysis of hybrid-RAMIE procedures, the learning curves were subdivided into initial phase (cases 1–25), consolidation phase (cases 26–50), and expert phase (cases 51–76) for better significance testing as described above.

The results document significant improvement in hybrid-RAMIE procedures in the transition from the consolidation to the expert phase. Particularly, operative duration, postoperative hospital and ICU length of stay, and anastomotic leakage show clear progress. This reflects the consolidation and optimization of surgical workflows and perioperative care, contributing to higher efficiency and patient safety. Overall, this demonstrates a clear efficiency increase with stable safety parameters with increasing experience for hybrid-RAMIE.

#### Total RAMIE analysis

For the analysis of total RAMIE procedures, the learning curves were similarly subdivided into initial phase (cases 1–40), consolidation phase (cases 41–80), and expert phase (cases 81–121).

In summary, the learning curve shows a significant improvement in time-dependent parameters, with stabilization or decline starting at approximately 70 patients (ICU stays already starting at approximately 50 cases). Between the consolidation and expert phases, all continuous endpoints are significantly better: total duration, thoracic and abdominal duration, and hospital and intensive care unit length of stay (all *p* < 0.001); the late, slight increase in abdominal duration does not alter the phase comparison. For binary endpoints, no significant differences were found between phases 2 and 3; however, the graphs for severe complications (Clavien-Dindo ≥ 3b) and anastomotic leakage showed decreasing trends during the expert phase. Overall, the analysis demonstrates a pronounced learning effect of total RAMIE, particularly with regard to efficiency and resource utilization, with stable complication rates.

Direct comparison of expert-phase performance between hybrid-RAMIE and total RAMIE revealed no statistically significant differences in operative duration (420.3 ± 86.3 vs. 426.7 ± 82.0 min, *p* = 0.719), postoperative hospital stay (19.7 ± 9.9 vs. 24.0 ± 20.9 days, *p* = 0.640), or ICU stay (4.0 ± 7.1 vs. 6.0 ± 14.5 days, *p* = 0.096). Severe complications (Clavien-Dindo ≥ 3b), anastomotic complications, pneumonia, and 30-day mortality were also comparable between expert phases (all *p* > 0.05).

### Patient-specific factors influencing learning curve parameters

In our exploratory “training cases” analysis (Supplementary Material), higher BMI and higher preoperative N-stage were associated with longer total operative duration (+ 3.03 min per kg/m², *p* = 0.04; + 23.75 min per stage, *p* = 0.01). Higher BMI was also associated with longer postoperative hospital stay (+ 0.65 days per kg/m², *p* = 0.02).

## Discussion

This analysis examines learning curves during our center’s transition from open to hybrid and robotic Ivor Lewis esophagectomy. The partially overlapping time periods reflect a gradual roll-out of new techniques, while established approaches remained in use depending on resources and case characteristics.

### Evolution: learning curve comparison across surgical epochs

Open esophagectomy plateaued at case 65 but showed deteriorating outcomes after case 70, suggesting asynchronous learning processes for operative performance versus postoperative management. Alternatively, this late deterioration may reflect era-related factors, including a shift in case-mix as minimally invasive programs expanded (e.g. less complex cases transitioning to minimally invasive approaches), leaving a comparatively higher-risk open cohort. A concurrent shift in institutional focus and resources during program transition may have further contributed, rather than operative performance alone.

The hybrid-open technique demonstrates a rapid technical learning curve with early stabilization of operational time and a plateau around case 15. At about the same time, clinical outcomes improve in key areas. Severe complications (Clavien–Dindo ≥ 3b) decrease from case 20 onward. Anastomotic leakage and pneumonia decrease around case 25. These patterns indicate a real gain in technical competence and improvements in perioperative management. The postoperative hospital stay remains fluctuating over the observation period with no clear directional trend. Despite an initial improvement, the postoperative ICU stay shows an overall upward trend from case 15 onward, with continued variability. The fact that complication rates decrease while ICU days increase and hospital length of stay shows no clear trend underscores the multidimensionality of the learning curve. A technical plateau does not automatically translate into uniformly shorter courses: postoperative processes and conditions likely influence outcomes, including staffing configurations with potential staff shortages, anesthesiology strategies, ICU admission criteria, and bed management. From around case 45, anastomotic leakage and pneumonia show a slight renewed increase, which may reflect greater case complexity or process variability and should be interpreted cautiously. Further, the limited number of cases (*n* = 52) should definitely not be ignored. This does not diminish the signals shown, but it does require cautious interpretation. As our larger case series in other techniques visually demonstrate, shorter observation periods could easily lead to premature assumptions of stabilization.

In RAMIE, performance improved significantly in the first few cases. Significant changes, beginning around case 77, coincide with the transition from hybrid-robotic to fully robotic approaches. This transition period explains the increase in individual endpoints—particularly postoperative hospital stays, intensive care unit length, and anastomotic leak rates—consistent with typical implementation effects, including new workflows, team training, and possibly more conservative postoperative management. Because this technical transition is reflected in multiple endpoints, our step to separate the analysis of the hybrid and fully robotic cases is highly advisable and useful. However, the temporary deterioration of postoperative parameters during the transition period, followed by renewed improvement, demonstrates that technological advances may lead to a temporary decline in performance before improved outcomes are achieved.

In summary, the hybrid-open technique shows earlier technical stabilization accompanied by parallel improvements in key complication rates, whereas open esophagectomy—despite achieving a technical plateau—tends to coincide with contemporary deterioration across multiple postoperative endpoints. RAMIE demonstrates early performance gains but exhibits a transient worsening of several outcomes during the transition from hybrid-robotic to fully robotic implementation. Across surgical epochs, learning curves proved multidimensional and partly asynchronous: intraoperative performance can improve early while postoperative outcomes evolve more slowly and heterogeneously. This multidimensional nature of learning curves necessitates separate monitoring of technical proficiency, outcome management, and complication prevention rather than relying on single parameters. Transitional phases may transiently worsen outcomes before benefits materialize. These findings support segmented, risk-adjusted monitoring and careful planning of technological transitions. Further, different case volumes must be considered: shorter series could favor premature plateau interpretation. To avoid premature conclusions about stabilization of performance, sufficient case numbers and longitudinal follow-up are essential.

### Robotics: learning curve comparison between hybrid-robotic and total robotic techniques

Regarding hybrid RAMIE, the data suggest a rapid increase in expertise with measurable efficiency gains from the consolidation to the expert phase, with broad onset of effects from cases 45–47. Total, abdominal, and thoracic operating times decrease significantly. At the same time, postoperative hospital and ICU stays are also shortened. Severe complications remain stable at a low level. The initially higher rate of anastomotic leakage in the consolidation phase and the subsequent decline in the expert phase are consistent with technical refinement and standardization of perioperative procedures. A clear downward trend in pneumonia is not evident, which could indicate, for example, independent influences from anesthesia, mobilization, or respiratory therapy.

With increasing experience, total RAMIE shows marked reductions in the time-dependent endpoints, which occur later overall, around case 70. The improvements affect total and partial operative times and are associated with shorter lengths of hospital and ICU stay. The binary safety endpoints do not differ statistically between the consolidation and expert phases but tend to show more favorable outcomes for major complications and anastomotic leakage in the expert phase. The slight late increase in abdominal time appears to represent expected variation and/or supervised teaching during a stable phase rather than systematic performance regression and does not alter the phase comparisons.

In direct comparison, both robotic strategies demonstrate significant efficiency gains with overall stable safety endpoints. Hybrid-RAMIE achieves improvements earlier, while total RAMIE begins later and then delivers consistent effects across multiple endpoints. When interpreting these learning curves, it is important to note that abdominal laparoscopy was already established in the hybrid-open era and was carried over unchanged into hybrid-RAMIE. The early effect observed with hybrid-RAMIE therefore likely reflects the continuity and standardization of the abdominal steps, whereas total RAMIE required an additional learning phase with the robotic abdominal phase. At the same time, it should be considered that the total robotic implementation benefited from previously gained hybrid experience, which presumably accelerated the learning process without fundamentally altering the subsequent improvement trend.

The fact that time-dependent metrics improve early and significantly, while binary outcomes remain largely stable and show only partial positive trends, again supports different timescales of technical learning and postoperative outcome development, as discussed above. Transition phases should therefore be actively managed – through proctoring, fixed team constellations, and simulation – to shorten temporary performance dips and reliably translate technical advances into clinical outcomes. Furthermore, the differing timescales of the learning curves indicate that the choice of robotic approach should be aligned with the institutional context. A fully robotic strategy introduces an additional learning step with the robotic abdominal phase and requires a longer learning curve before improvements become stably apparent across multiple endpoints. High case volumes and stable teams could shorten this start-up period and allow the approach to be fully leveraged. As previously mentioned, total RAMIE has been associated with better patient outcomes [9]. These reported outcome advantages may justify the additional complexity and resource investment required for total robotic implementation. However, the longer learning process and temporary performance degradation during the transition require clear inclusion criteria for early cases (e.g. low-risk profile), structured proctoring, standardized leak prevention and monitoring protocols, and established ICU admission and discharge criteria. Our exploratory predictor analysis (Supplementary Material) supports this approach, indicating that higher BMI and more advanced preoperative nodal stage are associated with longer operative duration. The prolonged learning curves identified in this analysis raise important volume and outcome considerations for clinical implementation: The extended learning trajectory may preclude achieving optimal outcomes within reasonable timeframes in centers with comparatively few cases per year. Therefore, the longer learning curve and resource expenditure should be weighed against the expected benefits. In such low caseload settings, a hybrid pathway may offer a more balanced trade-off between outcome goals and learning-curve demands. Minimally invasive procedures have proven advantageous in many areas, while open and hybrid techniques may still be important options for selected patients and institutional settings. In particular, the choice between hybrid and total robotic approaches should be volume-dependent.

The expert-phase comparison between hybrid-RAMIE and total RAMIE showed no statistically significant differences; however, subgroup sizes (*n* = 26 vs. *n* = 41) limit power to detect smaller effects, and the compared expert phases are not contemporaneous. Prior comparative work from our center has reported advantages for total RAMIE in selected outcomes [9]. Taken together, these data suggest that strong late-phase performance can be achieved with either approach, while potential incremental benefits of total RAMIE may be most apparent in larger cohorts and fully standardized settings.

Another important aspect is that our findings contradict the notion that RAMIE is “mastered” after several dozen cases. Recent systematic reviews highlight wide heterogeneity in RAMIE learning curves, with several studies indicating that initial proficiency may be achieved after comparatively few cases [14–17, 30–32], which should not be conflated with mastery. National data from the United States demonstrates continued efficiency improvements beyond 100 cases for robotic esophagectomy, with expert-level surgeons (101 + cases) showing significant reductions in operative times (-18% for esophagectomy) and substantial productivity gains [33]. Reliable assessment of inflection points and plateaus requires larger case volumes than many studies employ, particularly when technical transitions, team effects, or system changes play roles.

### Limitations and methodological considerations

This single-center study with surgeon-specific learning curves limits generalizability, though it minimizes inter-institutional variability. Open and hybrid-open procedures were performed by a different surgeon than robotic approaches, resulting in confounding of approach with surgeon identity and era. Consequently, between-era differences cannot be attributed solely to technique. The study’s longitudinal design introduces inherent confounding among surgical technique, surgeon experience, and institutional evolution. However, both surgeons received identical training from the same mentor within the same institutional framework and were experienced attending surgeons. Standardized institutional protocols governed all esophageal procedures. We therefore interpret findings as “institutional learning trajectories” encompassing technique, surgeon expertise, and protocol evolution. The mid-2018 organizational transition from department-specific to centralized interdisciplinary ICU management coincided temporally with RAMIE implementation, representing an ‘era effect’ where observed differences may reflect both surgical technique and evolving institutional infrastructure.

The RAMIE cohort’s more favorable baseline characteristics likely reflect evolving patient selection during program implementation. Risk-adjusted analyses suggest that shorter ICU and hospital stay remained associated with RAMIE after accounting for these differences, though residual surgeon and era effects remain. Across the 11-year period, outcomes were influenced by intertwined surgeon, era, and technique effects that cannot be fully disentangled in this observational design. While this precludes technique-only attribution, the within-cohort RAMIE trajectories (cases 1–197) demonstrate progressive stabilization of key time-dependent endpoints, supporting a learning-curve phenomenon within this institutional context.

CUSUM methodology detects performance changes but cannot distinguish between changes in intraoperative performance and external factors [27, 34, 35]. Different case acquisition rates may influence learning curve interpretation [27, 36], emphasizing the importance of adequate case volume for curve completion. The transition between robotic systems (da Vinci Si to Xi) coincided with technical approach changes, though surgical principles remained identical. There is no standardized definition of the learning curve in esophageal surgery, with studies operationalizing endpoints and phase boundaries heterogeneously, hampering comparability.

## Conclusion

Learning curves in esophageal surgery are multidimensional and partly asynchronous: operative performance tends to stabilise earlier, whereas postoperative outcomes mature more slowly and heterogeneously. In this institutional series, hybrid-robotic approaches were associated with earlier efficiency gains, whereas total RAMIE was associated with later-onset improvements across continuous endpoints (operative times and hospital/ICU stay), with binary outcomes remaining overall stable. In our total RAMIE cohort, stabilisation of time-dependent endpoints commonly required on the order of ~ 70 cases, arguing against fixed early-case cut-offs and favouring data-driven learning phase boundaries. Case volume is critical for improving both operative and postoperative outcomes, and adoption should be aligned with institutional context: full-robotic programs may be best suited for high-volume centres with stable teams, whereas hybrid pathways could offer a balanced option in lower-volume settings. When institutional capacity and team stability do not keep pace with the learning-curve timeline, transition phases may lengthen and consume additional resources while measurable outcome gains emerge later. Structured curricula with dedicated training and proctoring, coupled with risk-adjusted CUSUM monitoring and explicit management of transition phases, are essential to minimise performance dips and ensure patient safety. For early learning phases, a lower-risk patient selection is advisable.

## Supplementary Information

Below is the link to the electronic supplementary material.

Supplementary Material 1

## Data Availability

No datasets were generated or analysed during the current study.

## Abbreviations

ASA: American Society of Anesthesiologists
BMI: Body Mass Index
CROSS: ChemoRadiotherapy for Oesophageal cancer followed by Surgery Study
CUSUM: Cumulative Sum Control Chart
ECCG: Esophagectomy Complications Consensus Group
EndoVAC: Endoscopic Vacuum-Assisted Closure
FLOT: Fluorouracil, Leucovorin, Oxaliplatin, and Docetaxel
H-RAMIE: Hybrid Robot-Assisted Minimally Invasive Esophagectomy
ICU: Intensive Care Unit
RAMIE: Robot-Assisted Minimally Invasive Esophagectomy
T-RAMIE: Total Robot-Assisted Minimally Invasive Esophagectomy (full-RAMIE)
TNM: Tumor, Node, Metastasis (staging system)
WHO: World Health Organization
