# Supplementary material for: Learning curve analysis across three surgical eras in Ivor Lewis esophagectomy
Source: J Robot Surg. 2026 Mar 30;20(1):379. doi: 10.1007/s11701-026-03327-3 (PMC13035626; doi:10.1007/s11701-026-03327-3)
Supplement: Supplementary file 1 — Supplementary Material 1 [file 11701_2026_3327_MOESM1_ESM.docx]

**Supplementary Material**

**Patient-Specific Factors Influencing Learning Curve Parameters: Results**

The following analysis deliberately focuses on simplified, linearly modeled main effects of preoperative predictors. Effect estimates describe adjusted changes to be expected on average and should be interpreted as reference values. Potential interactions between predictors and nonlinear courses were not modeled to increase clinical applicability as practically as possible.

Regarding surgical duration, the average surgical duration was 41.36 minutes shorter in women (β = −41.36; p = 0.04). A higher BMI was associated with a longer surgical duration of +3.03 minutes per kg/m² (β = +3.03; p = 0.04). A higher preoperative N-stage was also associated with a prolongation of +23.75 minutes per stage (β = +23.75; p = 0.01). Regarding abdominal operative duration, higher BMI was associated with +2.18 minutes per kg/m² longer abdominal duration (β = +2.18; p = 0.02); each higher preoperative N-stage was associated with an increase of +13.60 minutes per stage (β = +13.60; p = 0.03). For thoracic operative duration, women showed, on average, 35.96 minutes shorter thoracic operative times (β = −35.96; p = 0.04). For postoperative hospital stay, BMI was positively associated with length of stay (+0.65 days per kg/m²; β = +0.65; p = 0.02). Among preoperative predictors, no significant association with ICU length of stay was observed (all p ≥ 0.05). Among preoperative predictors, no significant effect on severe complications (Clavien-Dindo ≥ 3b) was found (all p ≥ 0.05). Preoperative metastasis was associated with a markedly increased risk for anastomotic leakage (OR ≈ 12.0; β = +2.49; p = 0.01); higher Charlson Comorbidity Index increased the risk by approximately 30% per point (OR ≈ 1.30; β = +0.27; p = 0.03). Preoperative anticoagulation was associated with a markedly increased postoperative pneumonia risk (OR ≈ 5.68; β = +1.74; p = 0.01); preoperatively positive lymph node status increased the risk by approximately 20% (OR ≈ 1.20; β = +0.18; p = 0.04). The remaining preoperative patient characteristics described in the Methods section showed no significant associations with key learning curve parameters in our study (all p ≥ 0.05).

**Patient-Specific Factors Influencing Learning Curve Parameters: Discussion**

Analyses demonstrate consistent patterns: higher body mass index prolongs operative times and associates with longer hospital stays. Advanced preoperative N-stage links with longer procedure times, particularly in the abdominal component. Female patients demonstrate shorter operative times, pronounced in the thoracic phase. Anastomotic insufficiency risk increases with preoperative metastasis and higher comorbidity burden. Postoperative pneumonia shows associations with preoperative anticoagulation and positive lymph node status. No reliable associations emerged between preoperative factors and postoperative ICU duration or severe Clavien-Dindo complications.

These findings are clinically plausible. Increased body mass impedes exposure, dissection, and reconstruction [37, 38]. Higher N-stage requires extensive lymphadenectomy and complex dissection, consuming time. Gender likely serves as surrogate for differences in body habitus and tissue characteristics rather than serving as selection criterion. Increased anastomotic insufficiency risk with metastatic disease and high comorbidity aligns with compromised tissue quality and reduced reserves [39, 40]. The anticoagulation-pneumonia association likely reflects comorbidities and reduced respiratory reserve rather than direct medication effects.

For learning curves, preoperative characteristics shift inflection points and alter time to stabilization. Early phases benefit from reduced complexity case selection, including lower BMI, reduced N-stage, and minimal comorbidity. Higher-risk cases require structured approaches with senior supervision, standardized leak prevention protocols, and intensified pneumonia prophylaxis. Risk-adjusted monitoring prevents confounding technique effects with case-mix effects.

Methodologically, linear main effects were deliberately examined to provide simple, applicable guidance. Non-linear relationships and interactions remained unaddressed; effects should be interpreted as directional guidance. Future studies may examine interactions and threshold values while preserving pragmatic utility. The identified preoperative predictors (e.g., BMI, nodal status, metastatic disease) provide concrete guidance for case selection and pathway optimization.

| Table S1. | | | | |
| --- | --- | --- | --- | --- |
| Section A: Baseline Patient Characteristics | | | | |
|  | **Open (n=127)** | **Hybrid-Open (n=52)** | **RAMIE (n=197)** | **p value** |
| *Age (years), mean ± SD* | 61.7 ± 10.1 | 62.2 ± 10.3 | 64.7 ± 9.9 | 0.025 |
| *Male sex, n (%)* | 99 (78.0) | 44 (84.6) | 164 (83.2) | 0.407 |
| *BMI (kg/m²), mean ± SD* | 25.5 ± 5.1 | 25.8 ± 4.3 | 26.6 ± 5.3 | 0.261 |
| *WHO performance status, mean ± SD* | 1.4 ± 0.7 | 1.3 ± 0.7 | 0.8 ± 0.7 | < 0.001 |
| *WHO performance status 1, n (%)* | 9 (7.1) | 6 (11.5) | 71 (36.0) | < 0.001 |
| *ASA Score, mean ± SD* | 2.3 ± 0.6 | 2.3 ± 0.5 | 2.4 ± 0.6 | 0.046 |
| *Charlson Comorbidity Index, mean ± SD* | 4.2 ± 1.3 | 4.3 ± 1.4 | 5.1 ± 1.9 | < 0.001 |
| *Pretherapeutic N0, n (%)* | 20 (15.7) | 11 (21.2) | 92 (46.7) | < 0.001 |
| Section B: Risk-adjusted outcomes (RAMIE vs Open) | | | | |
|  | **Adjusted Effect*** | **95% CI** | **p value** | **R²** |
| *Operating time (min)* | β = +151.6 | +129.4, +173.7 | < 0.001 | 0.434 |
| *Postoperative hospital stay (days)* | β = −16.7 | −22.6, −10.7 | < 0.001 | 0.116 |
| *ICU stay (days)* | β = −15.2 | −19.6, −10.8 | < 0.001 | 0.169 |
| *Severe complications (CD ≥ 3b)* | OR = 0.51 | 0.24, 1.09 | 0.083 | 0.036† |
| Section C: Covariate effects in multivariable models (RAMIE vs Open) | | | | |
|  | **Coefficient (β)** | **95% CI** | **p value** |  |
| *Operating time (R²=0.434)* |  |  |  |  |
| Age (per year) | −0.9 min | −2.1, +0.3 | 0.129 |  |
| BMI (per kg/m²) | +4.0 min | +2.1, +5.8 | < 0.001 |  |
| WHO status (per level) | +28.1 min | +14.2, +41.9 | < 0.001 |  |
| Charlson Comorbidity Index (per point) | +6.1 min | −1.0, +13.1 | 0.093 |  |
| *Postoperative hospital stay (R²=0.116)* |  |  |  |  |
| Age (per year) | +0.3 days | −0.0, +0.6 | 0.063 |  |
| BMI (per kg/m²) | +0.6 days | +0.1, +1.1 | 0.013 |  |
| WHO status (per level) | −0.7 days | −4.4, +3.0 | 0.722 |  |
| Charlson Comorbidity Index (per point) | +0.3 days | −1.6, +2.2 | 0.726 |  |
| *ICU stay (R²=0.169)* |  |  |  |  |
| Age (per year) | +0.2 days | −0.0, +0.4 | 0.094 |  |
| BMI (per kg/m²) | +0.4 days | +0.1, +0.8 | 0.017 |  |
| WHO status (per level) | +0.6 days | −2.2, +3.3 | 0.684 |  |
| Charlson Comorbidity Index (per point) | +0.7 days | −0.7, +2.1 | 0.333 |  |
| *BMI* body mass index, *WHO* World Health Organization, *ASA* American Society of Anesthesiologists,  *ICU* Intensive Care Unit, *CD* Clavien-Dindo, *CI* Confidence Interval, *OR* odds ratio | | | | |
| *Section A: Baseline characteristics compared using Kruskal-Wallis (continuous) or Chi-square (categorical). N0 = no positive lymph nodes on pre-operative imaging. This reflects clinical staging at surgical decision-making. The 3-fold difference in N0 rate (RAMIE 46.7% vs Open 15.7%, p < 0.001) and 5-fold WHO status 0 difference (36.0% vs 7.1%, p < 0.001) indicate substantial baseline selection bias requiring statistical adjustment.*  *Section B: Multivariable regression (RAMIE vs Open) adjusting for age, BMI, WHO status, Charlson Comorbidity Index (n=324). *β = adjusted difference (negative favors RAMIE); OR < 1 favors RAMIE. †Pseudo R². Risk-adjusted analyses confirm RAMIE benefits persist after controlling for favorable baseline.*  *Section C: Patient-specific predictors from same models as Section B. Three separate models (one per outcome); all predictors entered simultaneously. β shows independent effect holding others constant.* | | | | |

| Table S2. “Expert Phase” Comparison between hybrid-RAMIE vs total RAMIE | | | |
| --- | --- | --- | --- |
|  | **hybrid-RAMIE “expert phase” (Cases 51-76, n=26)** | **total RAMIE “expert phase” (Cases 81-121, n=41)** | **p value** |
| *Operating time (min)* | 420.3 ± 86.3 | 426.7 ± 82.0 | 0.719 |
| *Postoperative hospital stay (days)* | 19.7 ± 9.9 | 24.0 ± 20.9 | 0.640 |
| *ICU stay (days)* | 4.0 ± 7.1 | 6.0 ± 14.5 | 0.096 |
| *Severe Complications (CD ≥ 3b), n (%)* | 3 (11.5) | 5 (11.9) | 1.000 |
| *Anastomotic leak, n (%)* | 2 (7.7) | 7 (16.7) | 0.465 |
| *Pneumonia, n (%)* | 6 (23.1) | 3 (7.1) | 0.076 |
| *30-day Mortality, n (%)* | 0 (0.0) | 0 (0.0) | 1.000 |
| *ICU* Intensive Care Unit, *CD* Clavien-Dindo | | | |
| *"Expert phases" defined by CUSUM stabilization (hybrid-RAMIE: cases 51-76; total RAMIE: cases 81-121). Mann-Whitney U test for continuous variables; Fisher exact for categorical. The absence of statistically significant differences in this expert-subset comparison (n=26 vs n=41) should be interpreted in context of the overall 197-case RAMIE experience and prior institutional publication (Hoelzen et al., J Clin Med 2023) demonstrating advantages for total-robotic approach. Small sample sizes in expert-phase subsets limit statistical power to detect differences.* | | | |
